# Microfluidic Synthesis of Magnetite Nanoparticles for the Controlled Release of Antibiotics

**DOI:** 10.3390/pharmaceutics15092215

**Published:** 2023-08-27

**Authors:** Cristina Chircov, Iulia Alexandra Dumitru, Bogdan Stefan Vasile, Ovidiu-Cristian Oprea, Alina Maria Holban, Roxana Cristina Popescu

**Affiliations:** 1 Department of Science and Engineering of Oxide Materials and Nanomaterials, National University of Science and Technology Politehnica Bucharest, 011061 Bucharest, Romania; cristina.chircov@yahoo.com; 2 National Research Center for Micro and Nanomaterials, National University of Science and Technology Politehnica Bucharest, 060042 Bucharest, Romania; bogdan.vasile@upb.ro (B.S.V.); ovidiu.oprea@upb.ro (O.-C.O.); 3 Faculty of Engineering in Foreign Languages, National University of Science and Technology Politehnica Bucharest, 060042 Bucharest, Romania; iulia.dumitru2212@stud.fim.upb.ro; 4 Research Center for Advanced Materials, Products and Processes, National University of Science and Technology Politehnica Bucharest, 060042 Bucharest, Romania; 5 National Research Center for Food Safety, National University of Science and Technology Politehnica Bucharest, 060042 Bucharest, Romania; 6 Department of Inorganic Chemistry, Physical Chemistry and Electrochemistry, National University of Science and Technology Politehnica Bucharest, 1-7 Polizu Street, 011061 Bucharest, Romania; 7 Microbiology and Immunology Department, Faculty of Biology, Research Institute of the University of Bucharest, University of Bucharest, 060101 Bucharest, Romania; alina_m_h@yahoo.com; 8 Faculty of Medical Engineering, National University of Science and Technology Politehnica Bucharest, 1-7 Polizu Street, 011061 Bucharest, Romania; 9 Department of Life and Environmental Science, National Institute for R&D in Physics and Nuclear Engineering Horia Hulubei, 30 Reactorului, 077125 Magurele, Romania

**Keywords:** magnetite nanoparticles, microfluidics, antibiotics, drug delivery systems

## Abstract

Magnetite nanoparticles (MNPs) have been intensively studied for biomedical applications, especially as drug delivery systems for the treatment of infections. Additionally, they are characterized by intrinsic antimicrobial properties owing to their capacity to disrupt or penetrate the microbial cell wall and induce cell death. However, the current focus has shifted towards increasing the control of the synthesis reaction to ensure more uniform nanoparticle sizes and shapes. In this context, microfluidics has emerged as a potential candidate method for the controlled synthesis of nanoparticles. Thus, the aim of the present study was to obtain a series of antibiotic-loaded MNPs through a microfluidic device. The structural properties of the nanoparticles were investigated through X-ray diffraction (XRD) and, selected area electron diffraction (SAED), the morphology was evaluated through transmission electron microscopy (TEM) and high-resolution TEM (HR-TEM), the antibiotic loading was assessed through Fourier-transform infrared spectroscopy (FT-IR) and, and thermogravimetry and differential scanning calorimetry (TG-DSC) analyses, and. the release profiles of both antibiotics was determined through UV-Vis spectroscopy. The biocompatibility of the nanoparticles was assessed through the MTT assay on a BJ cell line, while the antimicrobial properties were investigated against the *S. aureus*, *P. aeruginosa*, and *C. albicans* strains. Results proved considerable uniformity of the antibiotic-containing nanoparticles, good biocompatibility, and promising antimicrobial activity. Therefore, this study represents a step forward towards the microfluidic development of highly effective nanostructured systems for antimicrobial therapies.

## 1. Introduction

One of the greatest challenges that concerns society in the 21st century is represented by the increasing occurrence of microbial infections and the development of bacterial mechanisms to resist the activity of conventional antibiotics [1,2,3,4]. In this regard, the incidence of nosocomial infections represents a major mortality cause among patients, with severe socio-economic and ecological implications [2,5,6].

Therefore, current research trends focus on the development of alternative antimicrobial systems that could potentiate the effectiveness of antibiotics. Nanoparticles possess promising potential in terms of their use as antimicrobial agents due to intrinsic antimicrobial properties, as well as the possibility of being used as drug delivery carriers for targeted delivery [7,8,9,10]. Among them, magnetite nanoparticles (MNPs) are one of the most intensively studied types of nanoparticles owing to their unique functional and biological properties. Specifically, besides their intrinsic antimicrobial properties which makes them more suitable than polymeric nanoparticles, their unique magnetic properties allow for the direct targeting of the disease site using a magnetic field [11]. Therefore, MNPs are used in a wide variety of biomedical applications, ranging from antimicrobial and anticancer treatment alternatives [4,12,13,14,15,16] to diagnosis and imagining applications [17,18,19]. Nevertheless, the synthesis of MNPs generally involves the co-precipitation of iron ions into an alkaline medium, which poses several disadvantages in terms of control over the outcome properties of the nanoparticles, especially regarding their size and shape uniformity [20,21,22,23]. Therefore, different synthesis techniques that could overcome such limitations while maintaining the advantages of low cost, ease of application, and efficiency are required. Examples of non-conventional MNP synthesis techniques include the solvothermal [15,24,25,26,27] or microwave-assisted hydrothermal [13,28,29,30,31] methods, which allow for the control of the particle size by aging time variations, and the microfluidic approaches [32,33,34,35], through which particle size is controlled by varying the microchannel diameters, the flows within the microchannels, and the concentrations of the solutions. In this context, microfluidics has emerged as a promising alternative for obtaining nanomaterials with significantly narrow size distributions and uniform shapes and functional properties [23,36,37,38,39].

In this manner, the design of the present study focused on the development of streptomycin- and neomycin-loaded MNPs through the microfluidic synthesis method, which would enhance the uniformity of the nanosystems in terms of size, shape, polydispersity, surface reactivity, and drug loading. The obtained results confirm the potential of microfluidic approaches in the pathway towards their application for obtaining standardized nanoparticle-based drug delivery systems.

## 2. Materials and Methods

### 2.1. Materials

Ferric chloride hexahydrate (FeCl_3_·6H_2_O), ferrous sulphate heptahydrate (FeSO_4_·7H_2_O), sodium hydroxide (NaOH), streptomycin sulfate, and neomycin trisulfate were purchased from Sigma-Aldrich Merck (Darmstadt, Germany) and used as acquired.

The biocompatibility assay involved the use of normal BJ human dermal fibroblast cells (CLS, Heidelberg, Germany).

For the antimicrobial assays, three microbial strains were used (a Gram-negative bacterial species, i.e., *Pseudomonas aeruginosa* ATCC 27853, a Gram-positive bacterial species, i.e., *Staphylococcus aureus* ATCC 25923, and a fungal species, i.e., *Candida albicans* ATCC 10231), which were obtained from the Faculty of Biology, University of Bucharest.

### 2.2. MNP Synthesis

Both pristine and antibiotic-loaded MNPs were synthesized according to the procedures described in our previous studies [32,33]. Specifically, the nanoparticles were obtained through a microfluidic method involving the co-precipitation of iron ions using a lab-on-chip device [32,33]. The precursor stock solution was prepared by dissolving FeCl_3_·6H_2_O and FeSO_4_·7H_2_O in a 1:2 molar ratio at the final mass concentration of 1%. Subsequently, NaOH was dissolved at a 1 M concentration, followed by the addition of 1, 5, and 10% streptomycin sulfate or neomycin trisulfate. The obtained solutions were simultaneously introduced into the microfluidic device using a peristaltic pump equipped with four channels. The precursor solution was injected through the central inlet at the flow of 30 rpm, while the precipitating solution containing the antibiotics was administered through the side inlets at a flow of 15 rpm each. The obtained nanoparticles were collected, washed with deionized water until a neutral pH, and dried overnight at 40 °C (Table 1).

### 2.3. Morpho-Structural Characterization

#### 2.3.1. X-ray Diffraction (XRD)

The structural features of all samples were investigated using a CuKα radiation-provided PANalytical Empyrean diffractometer (PANalytical, Almelo, The Netherlands). Diffractograms were acquired between the 2θ angle values of 20 and 80°, with a 0.0256° step size and 1 s time per step. Further, the HighScore Plus software (version 3.0, PANalytical, Almelo, The Netherlands) was used for the Rietveld fitting of the acquired diffractograms (goodness of fit < 4 was considered acceptable) in order to determine the unit cell parameters, the average crystallite size, and the crystallinity of each sample.

#### 2.3.2. Transmission Electron Microscopy (TEM), High-Resolution TEM (HR-TEM), and Selected Area Electron Diffraction (SAED)

Sample preparation involved the dispersion of a small amount of nanoparticles into deionized water and placing 10 µL of the suspension into a 400-mesh lacey carbon-coated copper grid. The TEM and HR-TEM images and the SAED patterns were acquired on a high-resolution 80–200 TITAN THEMIS transmission microscope (purchased from FEI, Hillsboro, OR, USA). The microscope was equipped with a column EDXS detector and an image corrector, and it was operated in transmission mode at a voltage of 200 kV. The obtained images were used for the subsequent assessment of particle size distribution by measuring 100 nanoparticles within the ImageJ software (version 1.8.0, University of Wisconsin, Madison, WI, USA).

#### 2.3.3. Dynamic Light Scattering (DLS), Polydispersity Index (PDI), and Zeta Potential

In this study, 5 mg of the pristine and antibiotic-loaded MNPs was added to 15 mL of PBS 1x solution with a pH of 7.4 and dispersed using a Sonorex Digitec DT 514 ultrasonic bath (Bandelin, Berlin, Germany) for 10 min at 25 °C. A small amount of the dispersion was further introduced into the measurement cell and placed inside the DelsaMax Pro equipment (Backman Coulter, Brea, CA, USA). Three measurements were performed for each sample.

#### 2.3.4. Fourier Transform Infrared Spectroscopy (FT-IR)

IR spectra in the 4000–400 cm^−1^ wavenumber range were acquired for all samples in order to assess the functional groups present and, consequently, to demonstrate the presence of the antibiotics within the drug delivery systems. A mercury cadmium telluride detector-provided Thermo Scientific Nicolet iS50 (Thermo Fischer Scientific, Waltham, MA, USA) spectrometer was employed. Measurements were performed in the attenuated total reflectance (ATR) mode. Acquisitions were made at room temperature, with a resolution of 4 cm^−1^ and 64 scans for each sample. The OmnicPicta software (version 8.2, Thermo Nicolet, Thermo Fischer Scientific, Waltham, MA, USA) was used for data processing.

#### 2.3.5. Thermogravimetry and Differential Scanning Calorimetry (TG-DSC)

The amount of loaded antibiotics was assessed through the TG-DSC analysis, using an STA TG/DSC Netzsch Jupiter 449 F3 equipment (Selb, Germany). All samples were heated from 20 to 900 °C at a heating rate of 10 K/min and in a 50 mL/min dynamic air atmosphere.

#### 2.3.6. UV–Vis Spectrophotometry

A Thermo Evolution 600 double-beam UV–Vis spectrophotometer (Thermo Fischer Scientific, Waltham, MA, USA) and a 1 cm optical path glass cuvette were used for the UV–Vis spectroscopy measurements. Measurements were performed using a fixed wavelength of 202 nm for both types of antibiotics in order to assess their release profiles. For this step, 100 mg of each antibiotic-loaded sample was placed inside a dialysis bag, followed by their immersion in 50 mL of PBS 1x solution (pH of 7.4). All samples were maintained at 37 °C. At specific time-points, 1 mL of the supernatant was collected and replaced with fresh PBS. Data were expressed as the amount of antibiotic released (mg).

### 2.4. Biological Evaluation

#### 2.4.1. Cell Viability and Proliferation

Normal BJ human dermal fibroblast cells were cultured at 37 °C, 5% CO_2_, and 90% humidity in 10% fetal bovine serum-supplemented Dulbecco’s Modified Eagle Medium (DMEM). The nanoparticles were sterilized using UV radiation overnight and then suspended in deionized water at a concentration of 5 mg/mL via ultrasonic dispersion. Cells were seeded at a concentration of 5000 cells/well in 96-well plates and incubated for 24 h to allow cell attachment. Subsequently, the culture medium was replaced with nanoparticle-containing culture medium at a concentration of 0 and 50 µg/mL and incubated for 24 h. The MTT tetrazolium salts assay was employed for cell viability and proliferation investigations. Specifically, the nanoparticle medium was removed from the cells after the incubation period, and replaced with an MTT solution in complete culture medium and incubated for 2 h. This solution was prepared by dissolving 10% MTT (5 mg/mL in PBS) in complete culture medium. The method involves the ability of cells to metabolize MTT into formazan, which is proportional to cell viability. After the incubation time, the MTT culture medium was removed, and the formazan crystals were solubilized with DMSO. The amount of formazan produced is spectrophotometrically determined by measuring the absorbance at a wavelength of 570 nm. Blank samples, i.e., nanoparticles without cells, at the investigated concentrations, whose absorbance was subtracted from that of the samples with cells were used for all quantitative determinations. For each sample, three experiments were carried out. The obtained values were related to the negative control samples (which was assigned a value of 100%) in order to calculate the cell viability and differentiation ability. Cell viability and differentiation ability values were expressed as ±SEM (standard error of the mean).

Furthermore, the statistical significance was evaluated by the Student *t*-test function, with three levels of significance assigned (i.e., * *p* < 0.05, ** *p* < 0.01, and *** *p* < 0.001)

#### 2.4.2. Antimicrobial Activity

The antimicrobial assays performed are in accordance with previous studies [12,13,40,41]. All samples were sterilized using UV radiation for 20 min, followed by their dispersion in sterile deionized water at a concentration of 2 mg/mL.

An adapted diffusion test from the Clinical & Laboratory Standards Institute (CLSI) guidelines was employed for assessing the inhibition zone diameter. Briefly, Petri dishes containing the Mueller–Hinton agar medium for the *S. aureus* and *P. aeruginosa* bacterial strains and Sabouraud Dextrose broth for *C. albicans* yeast were used for the swab-inoculation of 1–3 × 10^8^ CFU/mL microbial suspensions (equivalent to 0.5 McFarland density standard). 10 µL of each nanoparticle suspension was added on the inoculated plates and incubated for 24 h at 37 °C. Subsequently, the diameter of the inhibition zone was measured, and the results were expressed in mm.

To determine the minimum inhibitory concentration (MIC), the microdilution technique in 96-well plates was employed. Each sample was subjected to binary serial dilutions from 2 mg/mL to 0.015 mg/mL in the appropriate nutritive broth, and the plates were inoculated using microbial suspensions of ~10^6^ CFU/mL. After the incubation for 24 h at 37 °C, the MIC was assessed through the naked-eye analysis and the lowest nanoparticle concentration that visibly inhibited the growth of the microbial strains was considered [42].

## 3. Results

The pristine and antibiotic-loaded MNPs obtained through the microfluidic synthesis method were characterized in terms of their morpho-structural and physico-chemical properties through XRD, TEM, HR-TEM, SAED, FT-IR, and TG-DSC. Subsequently, the cytotoxicity of the obtained structures was evaluated through the MTT assay on the BJ cell line, followed by the assessment of their antimicrobial activity through the inhibition zone diameter and MIC assays.

**Figure 1:**
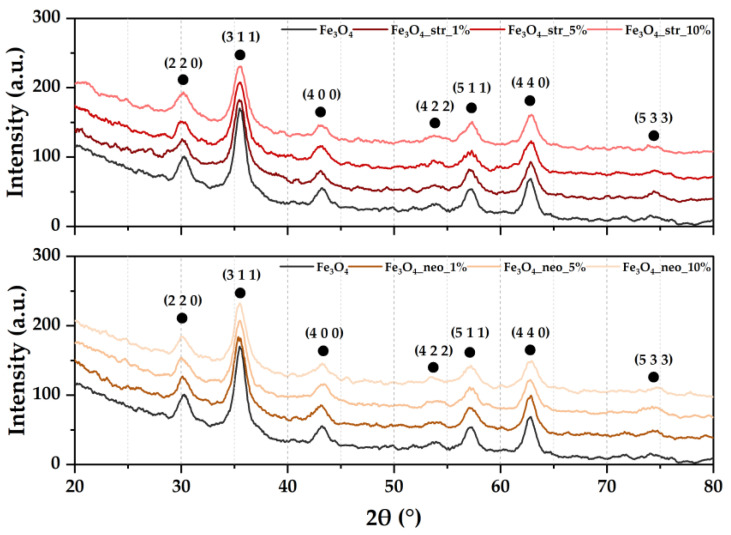
XRD patterns for the pristine and antibiotic-loaded MNPs and the associated Miller indices (•—magnetite).

XRD analysis was employed to determine the crystalline phases present within the samples (Figure 1). Within all samples, the XRD patterns reveal the presence of magnetite in the cubic crystallization system and the Fd3m space group as the unique crystalline phase through the characteristic Miller indices (according to JCPDS 01-084-2782 [43]). Therefore, the addition of antibiotics does not lead to the formation of secondary iron oxide phases, such as maghemite or hematite.

Furthermore, the acquired diffractograms were subjected to Rietveld refinement in order to determine the unit cell parameters, the average crystallite sizes, and the crystallinity of the samples (Table 2). Results show that the addition of the streptomycin antibiotic gradually decreases the average crystallite size up to the 5% concentration, while the addition of neomycin, up to the 10% concentration. Introducing the antibiotic also influences the crystallinity of the samples, which is inversely proportional to the antibiotic concentration. Nonetheless, the crystallinity of the samples with the highest antibiotic concentration is similar to the pristine nanoparticles. Therefore, it is safe to assume that antibiotic loading does not negatively affect the structural properties but rather acts as an adjuvant in controlling the crystallinity of the nanosystems.

**Figure 2:**
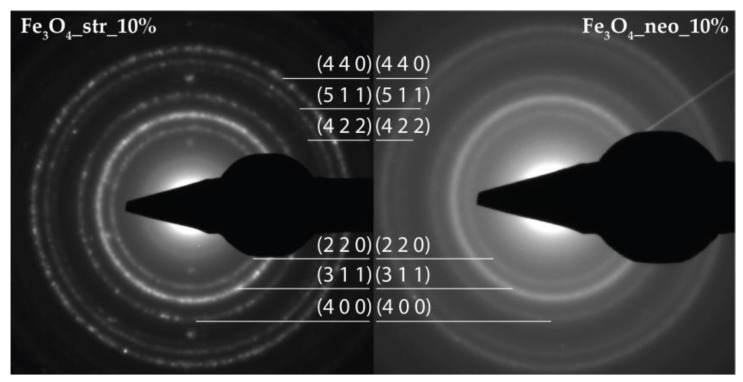
The SAED patterns for the Fe_3_O_4__str_10% and Fe_3_O_4__neo_10% samples.

Furthermore, the SAED results confirm the previous observations, as the diffraction rings within the patterns are associated with the Miller indices characteristic for magnetite (Figure 2). The neomycin-containing nanoparticles seem to have a lower degree of crystallinity, as the diffraction rings are more diffused, which is in accordance with the Rietveld refinement results.

**Figure 3:**
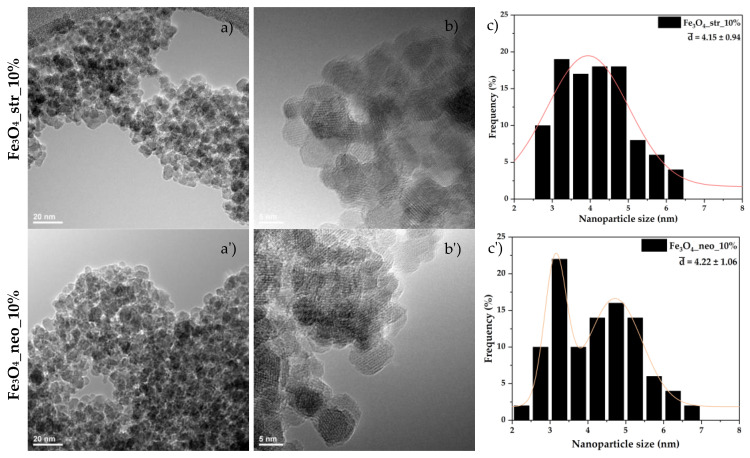
The TEM (**a**,**a′**), HR-TEM (**b**,**b′**), and size distributions (**c**,**c′**) for the Fe_3_O_4__str_10% and Fe_3_O_4__neo_10% samples.

The morphology of the nanostructures was assessed through TEM and HR-TEM (Figure 3). As can be observed, the nanoparticles are characterized by a quasispherical shape and an increased agglomeration tendency due to their surface energy. Furthermore, the high degree of crystallinity is demonstrated through the HR-TEM images, where the atomic planes within the nanoparticles are visible. The obtained images were further used to assess the size distribution of the nanosystems (Figure 3). The distributions are significantly narrow, between 2 and 7 nm, which further demonstrates the suitability of the microfluidic method for the synthesis of nanomaterials as it allows for dimensional control. There are no considerable differences between the two types of antibiotics regarding their effect on the morphology and size of the nanoparticles.

**Figure 4:**
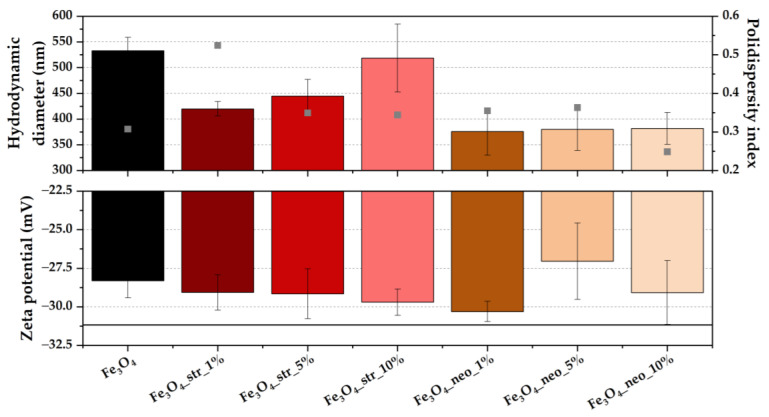
The hydrodynamic diameter (values shown as columns), the PDI (values shown as points), and the zeta potential values for the pristine and antibiotic-loaded MNPs (expressed as mean ± SD, *n* = 3).

The stability of the developed drug delivery systems was assessed through hydrodynamic diameter, PDI, and zeta potential measurements (Figure 4). As the results show, the hydrodynamic diameter of the nanoparticles decreases after antibiotic loading, which could be caused by the reduction of free hydroxyl groups present on the surface of pristine magnetite. In both cases, increasing the antibiotic amount further leads to proportionally higher hydrodynamic diameter values. Considering that the zeta potential also increases with the antibiotic amount, it could be assumed that the increasingly higher amount of antibiotics on the surface of the MNPs is the cause for higher particle diameters. The hydrodynamic diameter is larger in the case of streptomycin due to a higher degree of interaction with the solvent molecules. All PDI values are lower than 0.7, which generally indicates a narrow particle size distribution [44].

**Figure 5:**
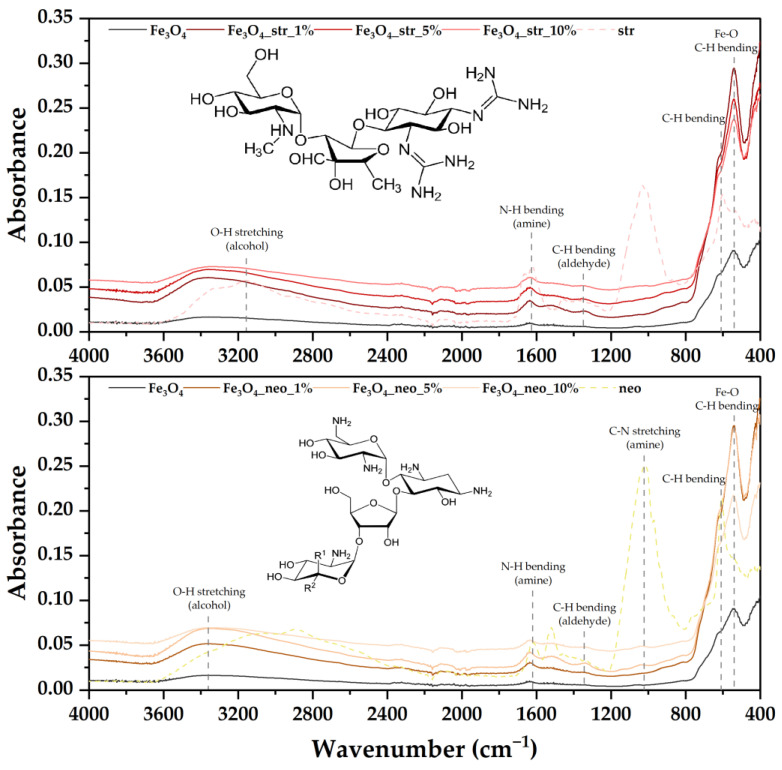
FT-IR spectra for the pristine and the antibiotic-loaded MNPs.

The efficiency of the antibiotic loading was further evaluated through the FT-IR analysis (Figure 5). The spectra show the Fe-O bond characteristic for magnetite at the 538 cm^−1^ wavenumber. As it overlaps with C-H bending, the increase in the maximum intensity with the addition of the antibiotics is attributed to the efficient loading of the drug molecules within the nanoparticles. Moreover, as there are no shifts of the maximum for the antibiotic-loaded samples, it is safe to assume that the antibiotics were chemically bound to the nanostructures through hydrogen bonding. The antibiotic loading can also be seen in the wavenumber range of 600–1800 cm^−1^, where absorption bands specific for the characteristic functional groups are present.

**Figure 6:**
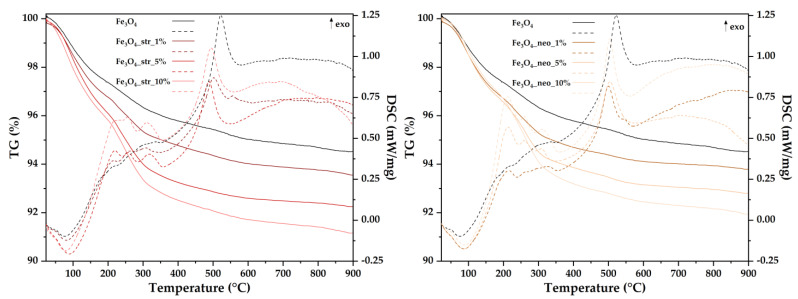
TG-DSC curves the pristine and the antibiotic-loaded MNPs.

The MNPs were further subjected to TG-DSC analysis in order to determine the mass loss and the associated thermal effects, which allowed for the estimation of the antibiotic loading within the nanoparticles (Figure 6, Table 3). It can be observed that up to 200 °C, all samples are losing residual water molecules, between 2.63 and 4.17%. The minimum solvent quantity can be found in the case of pristine MNPs, with larger quantities retained by the antibiotic-loaded samples. This process is accompanied by an endothermic effect on the DSC curve, with the minimum around 76–89 °C. Between 200 and 400 °C, the samples are losing mass, the processes being associated with some weak exothermic effects, indicating various oxidation reactions. The Fe^2+^ is oxidized to Fe^3+^ as the Fe_3_O_4_ (magnetite) is transformed to γ-Fe_2_O_3_ (maghemite) [45]. Concomitantly, the organic substances loaded onto the nanoparticles are being partially oxidized, as indicated by the multiple, different, small exothermic effects. After 400 °C, the small mass loss recorded can be assigned to the condensation of terminal –OH moieties but also to the burning of the residual carbonaceous mass. The strong, typical, exothermic effect around 500 °C is assigned to the transformation of maghemite to hematite [46,47]. The exothermic effect area increases slightly as the percentage of residual carbonaceous mass increases, from 111 to 122 J/g, a similar value being previously reported [46]. Furthermore, the antibiotic loading is proportional to the antibiotic concentration used for the MNP synthesis, with an increased loading efficiency for the streptomycin-loaded samples compared to the neomycin-loaded ones.

**Figure 7:**
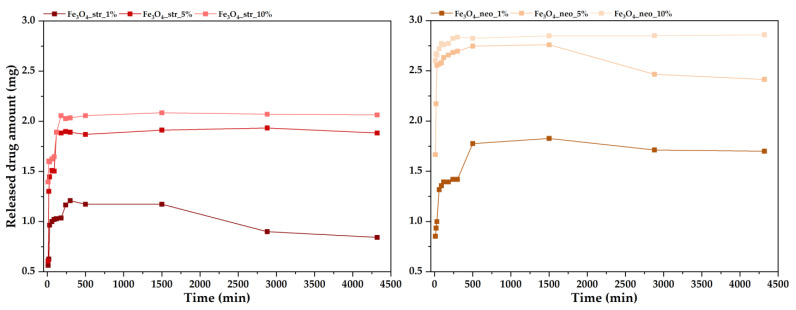
Drug release profiles for the antibiotic-loaded MNPs.

The drug release profiles were assessed through UV-Vis spectroscopy measurements (Figure 7). In both cases, the amount of released antibiotics is proportional to the concentration of drug loaded, with less significant differences between 5% and 10% than between 1% and 5%. Furthermore, it can be observed that the streptomycin antibiotic is gradually released from the nanoparticles (from ~0.6 to 2.2 mg), reaching a plateau after approximately 3 h that is maintained for the entire 72 h period. However, the drug amount released from the 1% sample decreases after 24 h, which could mean that the entire quantity is released within this timeframe. By contrast, the neomycin-loaded samples are characterized by a burst release that occurs within the first 10 min, reaching a plateau after approximately 6 h. In this case, the plateau is only maintained for 72 h for the 10% sample. In this manner, it could be concluded that the streptomycin-loaded samples provide a more controlled drug release owing to a higher encapsulation efficiency and a stronger interaction between the nanoparticles and the drug molecules.

**Figure 8:**
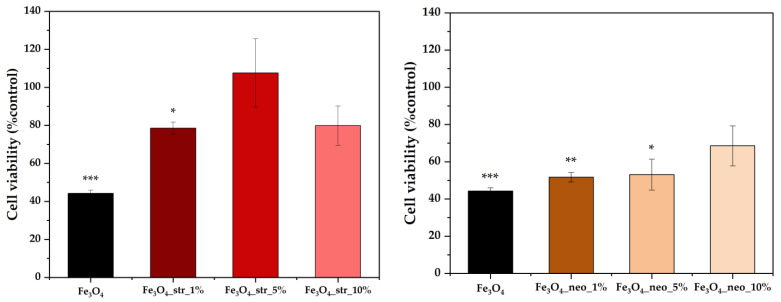
Cell viability values for the pristine and antibiotic-loaded MNPs (BJ cell line; values expressed as mean ± SD, *n* = 3; different signs indicate significant differences between the control and each sample; *—lower significance; ** and ***—higher significance).

Furthermore, the biocompatibility of the obtained drug delivery systems was determined through the MTT assay on the BJ cell line (Figure 8). On one hand, the cell viability characteristic for the pristine nanoparticles is significantly low, with values of approximately 40%. This effect could be attributed to the highly reduced nanoparticle size that could ensure their internalization within the cells and the production of reactive oxygen species. However, in both cases, the addition of the antibiotics within the nanostructured systems leads to an increase in cell viability, especially in the case of the streptomycin antibiotic. The highest cell viability is registered for the Fe_3_O_4__str_5% sample, for which the estimated drug load is around 2.40%. Increasing the streptomycin load to 3.55%, as it is in the case of the Fe_3_O_4__str_10% sample, leads to a decrease in cell viability due to possible toxic effects caused by the higher amount of the antibiotic. The presence of the neomycin antibiotic within the nanoparticles does not significantly increase the cell viability compared to the pristine sample, as was observed in the previous case, which could be attributed to the higher amount of drug released, as shown by the UV-Vis measurements.

In regard to their antimicrobial properties, the obtained drug delivery systems were subjected to the inhibition zone diameter and MIC assays. With respect to the inhibition zone diameter results (Table 4), it can be seen that the highest antibacterial efficiency was recorded against the *P. aeruginosa* strain, which is a Gram-negative bacterium, with a more pronounced effect registered for the streptomycin antibiotic. While both drugs are known to affect both Gram-positive and Gram-negative bacteria, streptomycin is mostly recommended for Gram-negative strains. Furthermore, the neomycin-loaded samples appear to be more efficient against Gram-positive bacteria, as the highest inhibition zones were measured against the *S. aureus* strain. The results against the *C. albicans* yeast further confirm the antimicrobial potential of the developed drug delivery systems, as the inhibition zone diameters are similar or even higher than the values measured for *S. aureus*. Nevertheless, the pristine MNP sample leads to inhibition zones similar to the antibiotic-loaded samples, thus demonstrating the intrinsic antimicrobial properties of MNPs primarily owing to the significantly small sizes. The MIC assay results (Table 5) confirm previous observations regarding the highest efficiency of the developed systems against the *P. aeruginosa* strain. Additionally, the antifungal effect is comparable to the effect against *S. aureus* bacteria.

## 4. Discussion

The present study aimed to develop a series of streptomycin/neomycin-loaded MNPs through a microfluidic approach that could further be employed in antimicrobial therapies. The obtained drug delivery systems were characterized by XRD, TEM, HR-TEM, and SAED, FT-IR, and TG-DSC in order to determine their physico-chemical and structural features. The biological evaluation of the obtained structures involved the assessment of their biocompatibility through the MTT assay on the BJ cell line and their antimicrobial effects through the inhibition zone diameter and MIC assays.

In regard to the structural properties of pristine MNPs, it can be seen that the average crystallite size and, consequently, the crystallinity of the nanoparticles is lower than those reported in our previous study, i.e., 6.71 nm and 12.91% and 8.06 nm and 18.53%, respectively [33]. Although the synthesis parameters were maintained constant (flow and concentration of the precursor and precipitating agent solutions), the difference between the two studies resides in the type of precipitator used. Therefore, it could be safe to assume that the use of NaOH as an alkaline agent leads to the formation of nanoparticles with lower crystallite sizes than NH_4_OH. One literature study focusing on this subject showed that the use of NaOH leads to an average nanoparticle size of 15.29 nm compared to 33.97 nm for nanoparticles obtained with NH_4_OH. This conclusion could be further translated to the crystallite size, as the nanoparticles appeared to be monocrystalline [48]. The mechanism behind this observation resides in the fact that the base amount and final pH are known to directly affect the nucleation and growth of the MNPs and, consequently, particle size. Specifically, it was shown that higher pH values lead to a decrease in nanoparticle size, which is in accordance with the present results [49]. Furthermore, as the antibiotics used for the development of the nanostructured drug delivery systems are alkaline compounds, the decreasing crystallite sizes with increasing concentrations are correlated with the previously described hypothesis. However, the increase in sample crystallinity with the addition of the antibiotic could be attributed to the contribution of the drug molecules to the crystallinity of the samples.

Furthermore, the higher drug loading determined for the streptomycin antibiotic could be attributed to a higher availability of the functional groups within the molecule to interact and form physico-chemical bonds with the hydroxyl groups present onto the surface of MNPs. A similar behavior was also observed in our previous paper, where the yield of sulfanilic acid functionalization was significantly lower than the yield for 4-sulfobenzoic acid [33].

To the best of our knowledge, this study represents the first one to investigate the synthesis and concomitant loading of drug molecules onto the surface of MNPs through a microfluidic approach. Nevertheless, there are other studies focusing on the microfluidic synthesis of MNPs [32,33,35,50,51], which have also obtained significantly narrow size distributions, which are similar to the ones described in the current study. Additionally, there are studies describing the direct synthesis of drug delivery systems through microfluidic devices, which mainly use polymers as the nanocarrier, such as curcumin-loaded shellac nanoparticles [52], liposomes containing plasmid DNA [53], poly(lactic-co-glycolic acid) particles loaded with indomethanic [54], doxorubicin [55], tamoxifen [55], or curcumin [56], 5-fluorouracil-loaded alginate–chitosan nanoparticles [57]. While these studies have reported higher encapsulation efficiencies, i.e., 93% [52], which could prove a higher suitability of using polymeric nanoparticles instead of inorganic nanoparticles, polymeric systems lack the intrinsic antimicrobial/anticancer properties that MNPs possess. This property was highlighted in the present study, as the pristine MNPs exhibited antimicrobial activities similar to the systems containing the antibiotics.

Therefore, the current study demonstrated the potential of microfluidic techniques for the development of drug delivery systems with significantly narrow size distributions. Additionally, the possibility to control and modulate the outcome properties of the systems, especially in regard to their crystallite and particle size and crystallinity, could consequently influence their biological behavior in contact with both cells and microbial species.

## 5. Conclusions

This study aimed to achieve one-step MNP synthesis and antibiotic loading through a microfluidic approach in order to obtain a series of drug delivery systems that could be applied in antimicrobial therapies. The results obtained demonstrated the presence of magnetite as the unique mineralogical phase and the formation of nanoparticles with significantly narrow size distributions and average nanoparticle sizes of ~4 nm. The results were in accordance with previously available studies, further proving the reproducibility of microfluidic approaches for nanoparticle syntheses. Furthermore, it was shown that the drug loading capacity is dependent upon the type of drug used, as the drug load calculated for streptomycin was higher than neomycin, especially at higher concentrations. The drug loading results were further translated into the biocompatibility assay, which showed cell viability results dependent upon the antibiotic type and concentration but also higher values for the antibiotic-loaded samples compared to the pristine ones. Furthermore, the antimicrobial activity was shown to be dependent upon the microbial strain; additionally, the pristine MNPs showed similar antimicrobial effects as the antibiotic-loaded ones. In this manner, this study successfully demonstrated the potential of the microfluidic method to obtain drug delivery systems that could be applied in antimicrobial therapies for both treatment and prevention of infections.

## Figures and Tables

**Table 1:** Summary of the obtained pristine and antibiotic-loaded MNPs.

Sample	Type of Antibiotic Used	Antibiotic Concentration (%)
Fe_3_O_4_	-	-
Fe_3_O_4__str_1%	streptomycin sulfate	1
Fe_3_O_4__str_5%		5
Fe_3_O_4__str_10%		10
Fe_3_O_4__neo_1%	neomycin trisulfate	1
Fe_3_O_4__neo_5%		5
Fe_3_O_4__neo_10%		10

**Table 2:** Unit cell parameters, average crystallite size, and crystallinity determined through Rietveld refinement.

Sample	Unit Cell Parameters		Average Crystallite Size ± Standard Deviation (SD) [nm]	Crystallinity [%]
	a = b = c [Å]	α = β = γ [°]		
Fe_3_O_4_	8.35	90	6.71 ± 0.46	12.91
Fe_3_O_4__str_1%	8.33	90	5.62 ± 0.57	15.54
Fe_3_O_4__str_5%	8.37	90	4.99 ± 0.10	14.62
Fe_3_O_4__str_10%	8.34	90	5.78 ± 0.66	12.84
Fe_3_O_4__neo_1%	8.34	90	6.23 ± 0.73	13.62
Fe_3_O_4__neo_5%	8.35	90	5.80 ± 0.38	13.20
Fe_3_O_4__neo_10%	8.37	90	5.46 ± 0.25	12.45

**Table 3:** The thermal effects, mass loss, and estimated antibiotic loading for the pristine and the antibiotic-loaded MNPs.

Sample	Mass Loss (%) 200 °C	Endo (°C)	Mass Loss (%) 200–400 °C	Mass Loss (%) 400–900 °C	Exo (°C)/ Area (J/g)	Estimated Load (%)
Fe_3_O_4_	2.63	76.8	1.58	1.27	521.9/111.6	-
Fe_3_O_4__str_1%	3.14	79.8	1.99	1.22	501.8/111.1	1.03
Fe_3_O_4__str_5%	3.87	88.4	2.85	1.00	488.4/112.0	2.40
Fe_3_O_4__str_10%	4.17	79.1	3.31	1.35	495.1/122.8	3.55
Fe_3_O_4__neo_1%	3.23	86.3	2.02	0.90	500.1/111.3	0.76
Fe_3_O_4__neo_5%	3.44	86.9	2.70	1.05	504.4/112.9	1.81
Fe_3_O_4__neo_10%	3.29	88.3	3.52	1.25	504.4/122.1	2.73

**Table 4:** Inhibition zone diameter measured for the pristine and the antibiotic-loaded MNPs.

Microbial Strain	Inhibition Zone Diameter (mm)						
	Fe_3_O_4_	Fe_3_O_4__str_ 1%	Fe_3_O_4__str_ 5%	Fe_3_O_4__str_ 10%	Fe_3_O_4__neo_ 1%	Fe_3_O_4__neo_ 5%	Fe_3_O_4__neo_ 10%
*S. aureus*	2	0	2	2	2	4	4
*P. aeruginosa*	7	6	6	6	6	5	6
*C. albicans*	5	2	4	4	5	4	5

**Table 5:** MIC values determined for the pristine and the antibiotic-loaded MNPs.

Microbial Strain	MIC (mg/mL)						
	Fe_3_O_4_	Fe_3_O_4__str_ 1%	Fe_3_O_4__str_ 5%	Fe_3_O_4__str_ 10%	Fe_3_O_4__neo_ 1%	Fe_3_O_4__neo_ 5%	Fe_3_O_4__neo_ 10%
*S. aureus*	2	2	1	2	2	2	1
*P. aeruginosa*	1	1	1	1	1	2	1
*C. albicans*	2	2	2	2	2	2	2

## Data Availability

Not applicable.
